# Intake of *Bifidobacterium longum* and Fructo-oligosaccharides prevents Colorectal Carcinogenesis

**DOI:** 10.5005/jp-journals-10018-1251

**Published:** 2018-05-01

**Authors:** Tadashi Ohara, Tatsuo Suzutani

**Affiliations:** 1 Department of Intestinal Bioscience and Medicine, Fukushima Medical University, Fukushima City, Fukushima, Japan; 2 Department of Microbiology, Fukushima Medical University, Fukushima City, Fukushima, Japan

**Keywords:** Colorectal carcinoma, Gastroenterology, Intestinal microbiome, Prebiotics, Probiotics, Short-chain fatty acids.

## Abstract

**Introduction:**

We aimed to investigate the effects of intake of yogurt containing *Bifidobacterium longum* (BB536-y) and fructo-oligosaccharides (FOS) in preventing colorectal carcinogenesis in healthy subjects, and the preventive effects of short-chain fatty acids (SCFA), whose production was enhanced by the intake of BB536-y and FOS, in human colon cancer cell lines.

**Materials and methods:**

The subjects were 27 healthy persons who were divided into a group taking yogurt containing BB536 (BB536-y group; n = 14) and a group taking yogurt containing BB536 and FOS (BB536-y with FOS group; n = 13) once a day for 5 weeks. The feces were sampled before and after the intake to analyze the amount of SCFA in the feces and the profile of intestinal flora, such as putrefactive bacteria and *Bacteroides fragilis* enterotoxin (ETBF). Subsequently, human colon cancer cell lines (DLD-1 cells, WirDr cells) were cultured in the presence of SCFA (butyric acid, isobutyric acid, acetic acid) in order to evaluate the cell growth-inhibitory activity of SCFA (WST-8 assay) by calculating the IC_50_ value from the dose-response curve.

**Results:**

Intake of BB536-y increased the total amount of SCFA in the feces and significantly suppressed the detection rate of ETBF and growth of putrefactive bacteria. Intake of BB536-y with FOS was associated with a higher *Bifidobacterium* detection rate than that of BB536-y alone. The contents of butyric acid, isobutyric acid, and acetic acid, namely, of SCFA, were also decreased. Analysis of the results of culture of DLD-1 cells and WirDr cells in the presence of butyric acid, isobutyric acid, and acetic acid revealed that each of the substances showed significant cell growth-inhibitory activity, with the activity being the highest for butyric acid, followed by that for isobutyric acid and acetic acid.

**Conclusion:**

These findings suggest that intake of both BB536-y and BB536-y with FOS prevents colorectal carcinogenesis.

**How to cite this article:** Ohara T, Suzutani T. Intake of *Bifidobacterium longum* and Fructo-oligosaccharides prevents Colorectal Carcinogenesis. Euroasian J Hepato-Gastroenterol 2018;8(1):11-17.

## INTRODUCTION

Patients with colorectal cancer have been reported to show decreased production of SCFA and increased production of putrefactive bacteria, and ETBF in the large bowel, which are attracting attention as risk factors for colorectal carcinogenesis.^1-3^ The probiotics BB536 has the effect of improving intestinal inflammation and eliminating ETBF.^4^ Fructo-oligosaccharides have been reported as prebiotics, with the potential to increase the intestinal density of *Bifidobacteria.^5^* Short-chain fatty acids generated by the intestinal flora have been reported to have the effect of inhibiting the production of putrefactive bacteria by acidification of the intestinal environment, and thereby of potentially controlling colon carcinogenesis.^1^

In this study, in order to investigate the effects of intake of BB536-y and FOS in preventing colorectal carcinogenesis, we examined the amounts of putrefactive bacteria, ETBF, and SCFA in fecal samples obtained from the BB536-y and BB536-y with FOS intake groups, and conducted culture of colon cancer cell lines in the presence of SCFA to study the cell-inhibitory effects of SCFA.

## MATERIALS AND METHODS

**Fig. 1:**
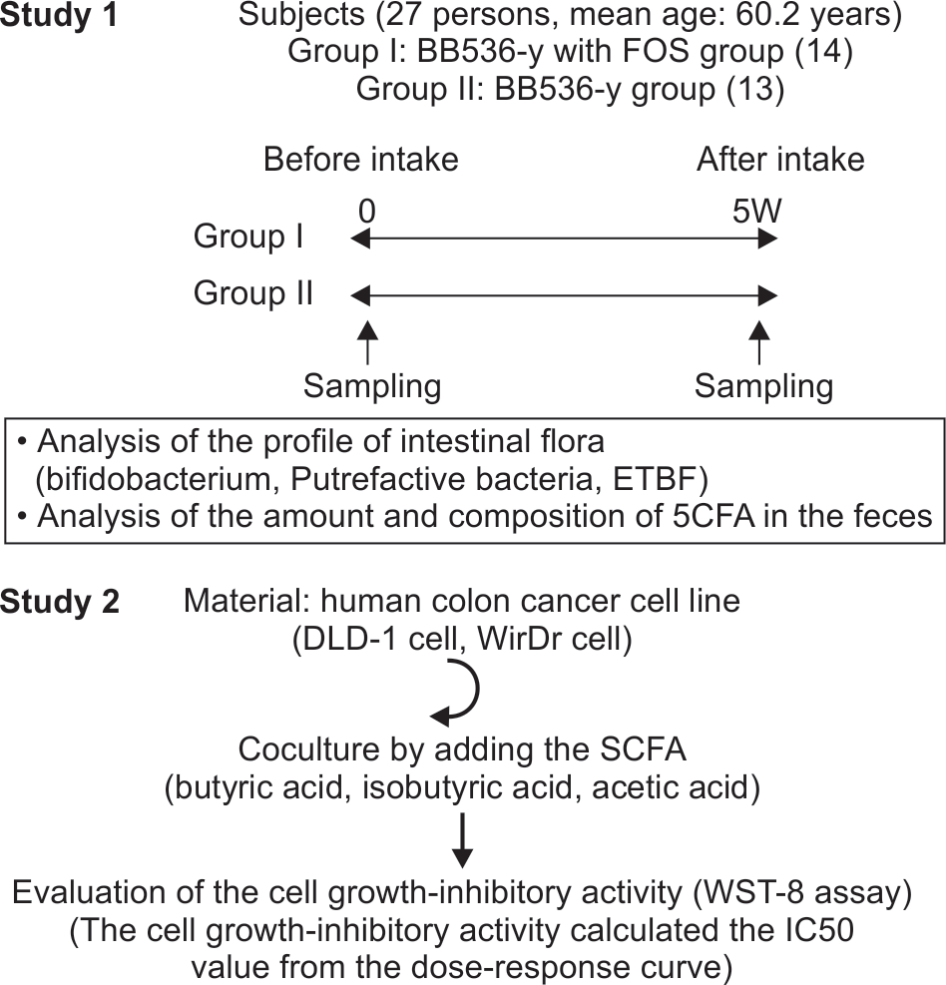
Experimental procedure

The subjects were 27 healthy persons (mean age: 60.2 years) who were divided into a group taking yogurt containing BB536 alone (BB536-y group; n = 14) and a group taking yogurt containing BB536-y and FOS (BB536 with FOS group; n = 13). The BB536-y or BB536-y with FOS (6 gm/day), depending on the group, was given to the subjects once a day for 5 weeks. The feces were sampled before and after the intake to analyze the amounts and composition of the SCFA, the profile of the intestinal flora, including the amounts of putrefactive bacteria and ETBF production (Fig. 1, Study 1).

### Microbial Community, SCFA, and pH Analysis

One gram of each fecal sample was placed immediately after collection in 9 mL of anaerobic diluent in a test tube, which was then tightly stoppered and stored at -80°C until the assay. Analysis of enteric bacterial flora was carried out in accordance with Mitsuoka’s method, but without using the plate-in-bottle technique, and the bacterial count per gram of wet stool specimen was expressed as the log-transformed value. Samples for measurement of the SCFA and pH were taken from the frozen fecal samples. After thawing, 0.5 gm of feces was diluted 5-fold with sterile distilled water and homogenized using a beads shocker (Yasui Kikai Corporation, Osaka, Japan) (2,500 rpm, 4°C, 2 min). The pH value was measured using a semiconducting electrode (ISFET pH meter KS701, Shindengen Electric Manufacturing Co., Ltd. Tokyo, Japan). One milliliter of the homogenate was transferred to a microcentrifuge tube. After centrifugation at 15,000g for 10 minutes at 4°C, 500 μL of supernatant was taken, and then 100 μL of 50 mM NaOH and 500 μL of chloroform were added into the supernatant. After centrifugation at 15,000g for 10 minutes at 4°C, 450 μL of the upper layer was collected and stored overnight at -80°C. Samples were defrosted and recentrifuged at 15,000g for 10 minutes at 4°C, and the supernatant (200 μL) was then filtered through a cellulose acetate filter (0.20 μm; DISMIC-13cp, Tokyo Roshi Kaisha, Ltd., Tokyo, Japan) (ADVANTEC). This supernatant was used to analyze the SCFA and other organic acids by high-performance liquid chromatography (HPLC). Eight organic acids were measured by HPLC (JASCO, Tokyo, Japan) equipped with two ShodexRSpak KC-811 columns (8 mm × 30 cm long; Showa Denko K.K., Tokyo, Japan), and a guard column (ShodexRSpak KC-G, Showa Denko K.K., Tokyo, Japan). The mobile phase used was 5% acetonitrile in 3 mM HClO_4_; the flow rate was 1.0 mL/min, and the column temperature was 55°C. The postcolumn reaction solution was composed of 0.2 mM bromothymol blue and 1.5 mM Na_2_HPO_4_/12H_2_O. The reaction solution flow rate was 1.5 mL/min. The detector was a multiwavelength detector set for detection at 430 nm (MD-1510; JASCO, Tokyo, Japan).

### *In vitro* Assessment of the Cell Growth-inhibitory Effect of SCFA (Butyric Acid, Isobutyric Acid, and Acetic Acid) against Human Colon Cancer Cells

Colon cancer cell lines (DLD-1, WirDr) were cultured in RPMI 1640, DMEN (containing 25 mM 4-(2-hydroxyethyl)-1-piperazineethanesulfonic acid) supplemented with 10% fetal bovine serum, 1% penicillin, and streptomycin, and maintained at 37°C in a humidified atmosphere containing 50 mL/L CO_2_. The cultured cell concentration was adjusted to 2 × 10^6^ cells per well (96-well plate), and Dulbecco’s phosphate-buffered saline was used as a vehicle for the test substance. As for changes in the pH of the cell culture medium by the addition of SCFA, it was ascertained that the pH of the cell culture medium was maintained at a constant value between 7 and 8 even after the addition of high concentrations of the test substances and that there was no intertest substance difference in the effects on the pH, and the influence of SCFA addition was examined by setting 11 graded concentrations (50 mM to 300 μM) of isobutyric acid and butyric acid and also 11 graded concentrations (250 to 1.5 mM) of acetic acid by serial dilution.

Similar to the experiment conducted in a previous study,^6^ human colon cancer cell lines (DLD-1, WirDr) were cultured in the presence of SCFA (butyric acid, isobutyric acid, acetic acid) and the IC_50_ values were determined to evaluate the cell growth-inhibitory activity of SCFA (Fig. 1, Study 2).

**Table 1:** **Table 1:** Results of fecal bacteria before and after intake

*Group*				*Before intake*		*5 weeks*	
I		Total bacteria		10.25 ± 0.60^a^		10.53 ± 0.81	
		*Bifidobacterium*		9.44 ± 0.11		9.80 ± 0.44^b^	
		Frequency of occurrence (%)		(9.7)		(30.6)*	
		*Clostridium perfringens*		7.42 ± 0.38		5.60 ± 0.65*	
		Frequency of occurrence (%)		(13.2)		(12.6)	
		*ETBF*		7.36 ± 0.85		1.90 ± 0.33*	
II		Total bacteria		10.35 ± 0.50		10.55 ± 0.61	
		*Bifidobacterium*		9.33 ± 0.21		9.74 ± 0.64	
		Frequency of occurrence (%)		(9.6)		(30.2)*	
		*C. perfringens*		7.40 ± 0.28		5.59 ± 0.45^*^	
		Frequency of occurrence (%)		(13.1)		(12.5)	
		*ETBF*		7.40 ± 0.79		1.80 ± 0.40*	

^a^Bacteria counts are expressed as mean ± SD of log 10 number per gram wet feces; ^b^Figures in parenthesis are frequency of occurrence (%); Statistically significant at *p < 0.05 levels.

**Table 2:** **Table 2:** Results of fecal putrefactive products before and after intake

*Group*		*Products*		*Before intake*		*5 weeks*	
I		Ammonia		52.20 ± 1.88^a^		44.20 ± 2.61*	
		Phenol		3.20 ± 6.66		2.15 ± 5.50	
		p-Cresol		112.22 ± 60.70		98.50 ± 53.86	
		Indole		12.01 ± 6.67		11.06 ± 7.00	
		Skatole		2.10 ± 2.01		2.20 ± 2.40	
		Sulfide		18.36 ± 5.52		12.60 ± 6.22*	
II		Ammonia		53.20 ± 1.78		43.20 ± 2.7i*	
		Phenol		3.29 ± 6.10		2.25 ± 6.50	
		*p*-Cresol		114.20 = 63.20		99.51 ± 57.96	
		Indole		12.51 ± 7.67		11.12 ± 7.10	
		Skatole		2.20 ± 2.11		2.20 ± 2.00	
		Sulfide		13.86 ± 6.12		12.57 ± 6.00*	

^a^Bacteria counts are expressed as mean ± SD of log 10 number per gram wet feces; Statistically significant at *p < 0.05 levels.

The cell growth-inhibitory activity was quantified by counting using a WST-8 assay kit (Dojindo Molecular Technologies, Inc., Japan), by measuring the spectropho-tometric absorbance (450 nm) of water-soluble formazan formed via reduction by intracellular dehydrogenase. The IC_50_ value, representing the test substance concentration, producing a 50% inhibition of cell growth, was calculated from the dose-response curve constructed by plotting the cell count of the control culture against the test substance concentration.

### Statistical Analysis

Data are presented as means of n experiments with the standard deviation (mean ± SD) and the standard error (mean ± SE). Statistical data processing was performed with XLSTAT (http://WWW.xlstat.com) using Student’s t-test. The p-values of less than 0.05 were considered as indicative of statistical significance.

## RESULTS

Both the BB536-y and BB536y with FOS intake groups showed an increase in the total amount of SCFA and suppression of the growth of putrefactive bacteria in the feces; ETBF could also hardly be detected in the feces in either group (Tables 1 and 2).

The *Bifidobacterium* detection rate was higher in the BB536-y with FOS group than in the BB536-y group (Table 1).

The butyric acid and isobutyric acid (SCFA) contents were significantly increased in the fecal samples; the acetic acid content was also increased, although the increase was not statistically significant (Table 3).

**Graphs 1A to D:**
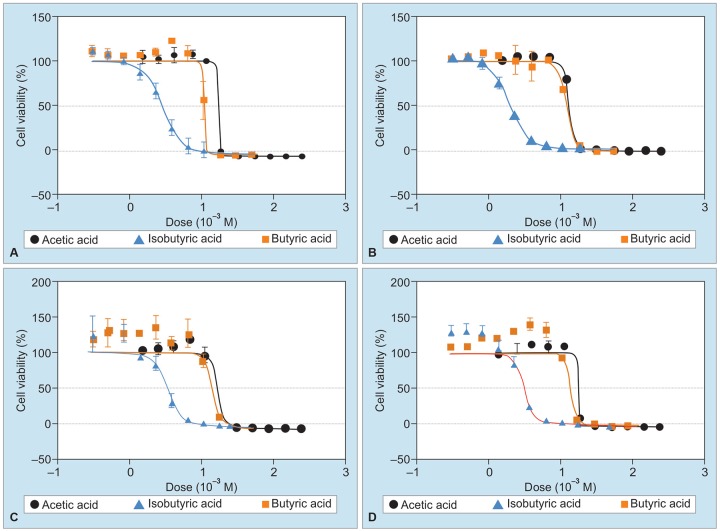
Volume response curves of the cell growth-inhibitory activity in DLD-1 cell

**Graphs 2A to D:**
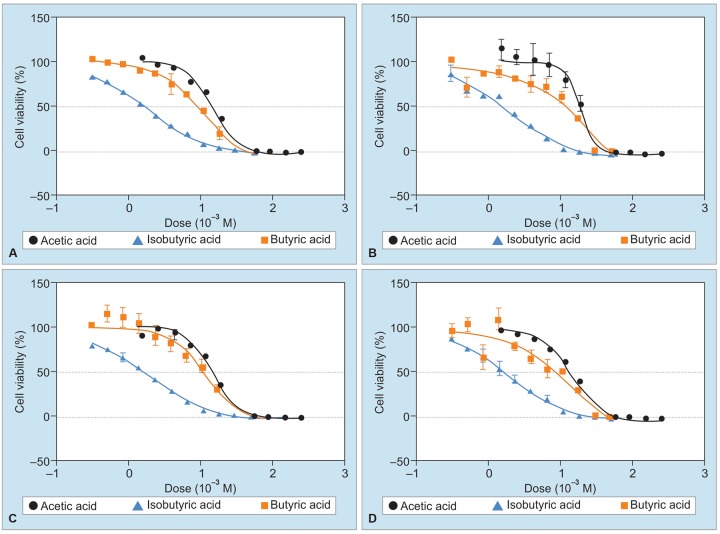
Volume response curves of the cell growth-inhibitory activity in WirDr cell

In the culture systems in which DLD-1 cells and WirDr cells were cultured in the presence of butyric acid, isobutyric acid, and acetic acid, each of the substances was found to exert significant cell growth-inhibitory activity, with the IC_50_ value being the highest for butyric acid, followed by that for isobutyric acid and acetic acid, in that order (Graphs 1, 2 and Table 4).

**Table 3:** **Table 3:** Results of fecal SCFA before and after intake

*Group*		*Fatty acid*		*Before intake*		*5 weeks*	
I		Lactic acid		1.42 ± 0.30^a^		1.90 ± 2.77	
		Propionic acid		2.20 ± 0.50		2.37 ± 0.69	
		Formic acid		2.18 ± 1.41		2.32 ± 1.19	
		Acetic acid		4.30 ± 1.00		4.33 ± 1.50	
		Butyric acid		1.58 ± 0.38		2.19 ± 0.80*	
		Isobutyric acid		5.98 ± 2.30		8.01 ± 4.40*	
		Valeric acid		0.47 ± 0.38		0.61 ± 0.38	
		Isovaleric acid		3.65 ± 1.30		4.48 ± 2.33	
		Total SCFA		21.70 ± 1.02		26.10 ± 1.80	
II		Lactic acid		1.40 ± 0.40		1.91 ± 2.66	
		Propionic acid		2.19 ± 0.30		2.36 ± 0.70	
		Formic acid		2.17 ± 1.38		2.33 ± 1.20	
		Acetic acid		4.32 ± 1.10		4.34 ± 1.60	
		Butyric acid		1.58 ± 0.38		2.21 ± 0.50*	
		Isobutyric acid		5.85 ± 2.30		8.11 ± 4.80*	
		Valeric acid		0.50 ± 0.36		0.62 ± 0.32	
		Isovaleric acid		3.77 ± 1.50		4.51 ± 2.43	
		Total SCFA		22.80 ± 1.11		27.19 ± 1.81	

^a^Bacteria counts are expressed as mean ± SD of log 10 number per gram wet feces; Statistically significant at *p < 0.05 levels.

## DISCUSSION

In recent years, it has been reported that the enteric bacterial flora (intestinal microbiota) is involved in the control of the host immune functions and synthesis of glucides and lipids, in the prevention of carcinogenesis.^7-9^ Control of the enteric bacterial floral balance (intestinal milieu) leads to prevention of various diseases by control of the underlying pathophysiological processes. Functional foods, such as probiotics, prebiotics, and synbiotics have received considerable attention as foodstuffs with the potential to dramatically improve the intestinal milieu.^10,11^ Prebiotics are sparingly digestible saccharides that take part in the growth of probiotics in the intestine by being taken up by probiotic bacterial species, while synbiotics are food ingredients containing probiotics and prebiotics in synergistic combinations.

Genetic carcinogenesis (familial aggregation carci-nogenesis) accounts for about 20 to 30% of all cases of colorectal cancer, while the remaining approximately 70 to 80% of cases arise as a result of so-called sporadic colorectal carcinogenesis, in which lifestyle/environmental factors, such as diet, physical exercise, and luxury grocery items play a major role.^12-14^ In regard to the diet, diminished dietary fiber intake results in a delay in the transit time of the contents of the large intestine by inducing increases in the fecal fatty acid and free fatty acid levels and reducing the fecal bulk. Consequent worsening of the intestinal milieu causes an increase in the growth of noxious bacteria and of bile acids in the intestine. The World Cancer Research Fund survey report on the relationship of diet, nutrition, and physical activities with colorectal carcinogenesis identified consumption of red meat and processed meat, habitual alcohol drinking, obesity, and a tall habitus as significant risk factors for colorectal carcinogenesis.^15^ The report also identified daily physical activity, and consumption of foods containing dietary fiber, milk, and calcium as positive preventive factors.^16-18^ Dairy products are rich in calcium content, and the incidence of colorectal cancer is known to be low in districts with high consumption levels of dairy products. It has also been reported that production of putrefactive bacteria in the intestines is suppressed in subjects with high intake of dairy products as compared with that in subjects with lesser intake of dairy products, which suggests that alterations in the intestinal milieu may have some impact on colorectal carcinogenesis.^1,19,20^ Two reports, referred to below, have been published so far on the relationship of the intestinal bacterial flora with colorectal carcinogenesis. According to the first, increased growth of *Clostridium perfringens* in the feces and an alkalotic stool pH were evident in patients with colorectal cancer, in whom facilitation of intestinal noxious bacteria and depression of intestinal peristalsis were noted.^3^ Depression of natural killer (NK) cell activity and decreased SCFA production were also evident in patients with colorectal cancer, which could be thought to suppress apoptosis and inhibit bacterial growth.^15-22^ Furthermore, it has been reported that bu-tyrate prevents colorectal carcinogenesis by controlling histone deacetylase.^23,24^ The mechanism of the antitu-mor action of SCFA diverges into many branches,^25-27^ and further examination is necessary. According to the other report, a study in an animal model of colon cancer revealed an increase in the level of ETBF in the feces, which caused persistent chronic inflammation due to destruction of the intestinal GAP junction with evidence of accelerated expression of such transcription factors as STAT-3, which has an antiapoptotic effect.^2,3^

**Table 4:** **Table 4:** Results of the cell growth-inhibitory activity after adding SCFA to colon cancer cell line

		*IC50 (mM, mean ± SEM, n = 4)*			
		*DLD-1 cell*		*WirDr cell*	
Isobutyric acid		12.9 ± 0.80		10.2 ± 0.77	
Butyric acid		2.89 ± 0.29		1.59 ± 0.04	
Acetic acid		16.6 ± 1.10		15.1 ± 1.25	

On the contrary, it has been reported that ingestion of a probiotics *(Lactobacillus gasseri)* produced dramatic improvement of the intestinal milieu in normal subjects.^1^ This implies that the ingestion of the probiotics resulted in a conversion of the pH of the intestinal contents to an acidotic pH range, thereby leading to suppression of the growth of putrefactive bacteria and increased SCFA production in the intestines. The report further described an augmentation of the host NK cell activity in response to ingestion of the probiotics.^1^ The study also demonstrated that augmentation of the NK cell activity served to inhibit tumor cell growth, and that SCFA inhibited cell growth via Wnt signaling_._^28-32^

There was no significant difference in the intestinal milieu-improving effect between a group of healthy subjects receiving BB536-y, i.e., yogurt containing *Bifidobacterium longum* (probiotics) alone and a group of healthy subjects receiving FOS, a sparingly digestible saccharide, administered in combination with BB536-y (probiotics) in the present study. In both groups, a shift of the fecal pH to the acidotic range, inhibition of putrefactive bacterial proliferation, and increased production of SCFA in the feces, and augmentation of NK cell activity were observed, reflecting a dramatic improvement of the intestinal milieu. Increase in the contents of SCFA, particularly those of butyric acid and isobutyric acid, was noted. Butyric acid and isobutyric acid, in particular, among the SCFA, have been reported to exert an inhibitory effect on colorectal carcinogenesis. The present results suggest that colorectal carcinogenesis can be inhibited by ingestion of functional foods, such as probiotics and prebiotics.

However, the important issue of human enteric bacterial flora varying among individuals needs to be borne in mind in the context of the results of this study.

These differences among individual persons may be attributable to differences in the intestinal commensal bacterial species (organic acid-utilizing organisms). In other words, probiotics take part in the production of SCFA from lactic acid in the intestinal tract via commensalism with intestinal commensal bacteria, and that the intestinal commensal bacterial species vary among individuals, resulting in differences in the compositions of the SCFA produced. Eventually, the effects of ingestion of functional foods, such as prebiotics, probiotics, and synbiotics vary among individuals, posing a major problem in the clinical application of these foods.

Major bacterial species currently used as probiotics are species of *Bifidobacterium* and *Lactobacillus.* Many problems remain to be resolved yet in relation to future clinical application of probiotics, and conducting large-scale clinical trials, examination of the behavior of probiotics in the intestinal tract, and identification of intestinal commensal bacteria that produce SCFA via commensalism with particular probiotic bacterial species are needed. Also, the development of new sparingly digestible saccharides (prebiotics) involved in probiotic bacterial growth, and of probiotic bacterial species and synbiotics, which specifically produce beneficial SCFA is needed. We believe that the present report will undoubtedly serve as a foundation for the development of new strategies for preventing colorectal carcinogenesis.
